# A sense of distance and movement characteristics of golfers tested without visual feedback of outcomes: Is a putt that feels subjectively good also physically good?

**DOI:** 10.3389/fspor.2022.987493

**Published:** 2022-10-25

**Authors:** Yumiko Hasegawa, Ayako Okada, Keisuke Fujii

**Affiliations:** ^1^Faculty of Humanities and Social Sciences, Iwate University, Morioka, Japan; ^2^Japan Ladies Professional Golfers' Association, Tokyo, Japan; ^3^Graduate School of Informatics, Nagoya University, Nagoya, Japan; ^4^RIKEN Center for Advanced Intelligence Project, RIKEN, Fukuoka, Japan; ^5^PRESTO, Japan Science and Technology Agency, Kawaguchi, Japan

**Keywords:** outcome estimation, self-assessment, sensorimotor feedback, mental representation, professional, kinematics, impact force, golf putting

## Abstract

For motor tasks that require fine-tuning, such as adjusting the applied force according to the distance to the target, as required for driving and putting in golf, it is important to develop a high degree of sensitivity of one's movement-produced feedback. In previous research focusing on mental representation in golf, this ability to control distance has been called “a sense of distance”. In particular, this study focused on three skills: motor control of the putter head, perception of the impact force, and prediction of the ball's travel distance. However, the relationship between the motor control of the putter head and the error of the outcome estimation is yet to be clarified. The purpose of the present study is two-fold: first, to clarify whether kinematic variation of putter head is correlated with error in estimating the outcome and, second, to quantitatively evaluate the performer's sensations of good and poor performance generated by the ball's impact, for a comparison of the kinematics and impact force of the putter head based on their assessment. Twelve professionals and 12 intermediate amateurs played two distance targets (at 2.4 and 4.8 m) without visual feedback of the outcomes. The kinematics of the putter head, impact force, final ball position, outcome estimation, and subjective assessment were measured. Our results show that the variability in the peak velocity was moderately correlated with the error of the outcome estimation in amateurs' 4.8-m putting task. In addition, amateurs estimated undershoots (overshoots) when they provided worse (better) evaluations. However, the amateurs' trials that were rated as better putts were actually overshoots. These results suggest that the subjectively “good putt” of amateurs was not physically good, and the amateurs putted hard to compensate for the risk of undershoots. However, no remarkable feature of the professional's sense of distance was found. This suggests that professional golfers' sensation is not significantly different from the outcomes that can be physically detected.

## Introduction

As long-term training develops skills, performers will be able to use internally generated information from movements to estimate the outcome of a task more accurately. In fact, we can see the appearance of athletes who are convinced of a successful outcome immediately after their performance in sport games. There are similar reports in scientific research, for example, as shown in a study by Gray, Beilock, and Carr (1), “As soon as the bat met the ball, I knew it was gone”. That is, the ability to predict the outcome of one's performance is not only based on one's skill level but also on how one is performing at a given moment (1). Sensitivity to feedback, generated by the movement to achieve a goal, is very important in sports, and a few studies have investigated the relationship between skill levels and the accuracy of estimating outcomes based on such sensations (1, 2). In the present study, we used the golf-putting task to investigate the sensitivity of golfers to distance control and their kinematic characteristics.

Previous studies have reported that the prediction of results by experts is more accurate than those of nonexperts. A study using a virtual batting task suggested that experts paid more attention to clues related to prediction of the outcome and that is why their predictions are accurate (1). The rationale for this suggestion was based on the common coding theory (3). According to Basevitch et al. (2), low-skilled players cannot estimate their outcomes accurately because they have not developed a refined and well-organized action representation. The theoretical framework that guided their study was the mental representations approach (4), in which performance is mediated by internalized mental representations and, with task-specific expertise, mental representations are acquired. To capture the underlying mental representation of the performer, Basevitch et al. (2) manipulated performers' visual information using a soccer passing task at two distances, and analyzed the kinematics of the performers. The results showed that high-skilled players performed significantly better than low-skilled players on the actual passing and estimation tasks, under different visual conditions, and mental representation and vision mediated their performance. Furthermore, they also suggested that the dependence of such mental representations depends on the type of the task. That is, for closed self-paced tasks, online visual information is less important and there's more dependency on mental representation, as the environment is relatively static during performances such as golf-putting.

Golf putting is a closed self-paced task that requires a high degree of sensitivity to its own movement-produced feedback. According to the closed-loop model for human performance, the feedback (i.e., muscle, movement, and environmental sensations) obtained by performing the task is matched against the reference produced when the movement was planned, and the difference is detected as an error (5). To adjust the force exerted on the putter-head, golfers may need to sense the tactile and kinesthetic cues transmitted through the club shaft (6–8). Further, the putting must generate the intended club-head velocity in the intended direction (9, 10).

In previous research focusing on mental representation in golf, this ability to control distance has been called “a sense of distance” (11). They assumed that the sense of distance may be learned and expressed through three separate skills: motor control of the putter-head, perception of the impact force, and prediction of the ball's travel distance. Thereafter, they conducted an experiment with high-level amateurs and novice golfers to investigate their sense of distance using these three movement variables for different distance targets (1.2, 2.4, and 3.6 m). In principle, if high-level golfers have less putter-head kinematic variability, they can cause the ball to travel to the intended distances, and their estimation of outcomes would be more accurate than that of novices (11). Thus, they assumed that the variability in putter-head swing kinematics would have a strong correlation with the error of outcome estimations. Their results showed that the movement variability of high-level amateurs was less than that of novices and their estimates were more accurate than those of novices. However, the expected correlation between the two variables was not confirmed. We believe that their hypothesis is reasonable based on previous research (1, 2). Therefore, we reexamine the relationship between the variability in putter head-swing kinematics and the error of outcome estimations as the first purpose of our study. We hypothesize that there is a positive correlation between the two variables. As considered in previous studies, we set and compare two distances (shorter distance; 2.4 m and longer distance; 4.8 m) because the difficulty of the task depends on the distance to the target (12). Also, previous studies suggested that the difficulty of the task influences the variability of the kinematic variables and the error of outcome estimations (2, 11). Tanaka and Iwami (11) focused on the impact force as a variable for measuring the sense of distance, but it was not actually examined. Thus, we added the impact force as the dependent variable.

Professionals show excellent putting performance on the actual golf course. Amateurs, on the other hand, hit putts that are too strong or too weak for the distance to the target. We presume this may be because amateurs have an immature sense of distance. Examining the average error of the outcome estimation and the actual average error from the target along with the golfer's subjectivity for the putt (good or poor), we may be able to get a better understanding of the characteristics of the golfer's sense of distance. The performer's subjectivity, such as good (success) or poor (failure), of the sense generated from their performance, should correspond to the rational movement and the accurate outcome in their learning process, especially in sports. It has been reported that attention to feedback, generated by one's movements, facilitates learning [e.g., Hogan and Yanowitz (13), Swinnen et al. (14)]. The results of the studies indicate an improvement in the match between the performer's senses and the accuracy of the physical results. In the case of golf putting, the success or failure of the plan to deliver the ball to the target (e.g., magnitude of force and length of time) is judged by the performer from the feedback obtained when the movement is performed. If the performer is relatively well-learned, the quality of the putting is assessed immediately (good or poor). By quantitatively evaluating the performer's subjectivity when hitting the ball, we believe that the characteristics of the golfer's sense of distance can be captured. To the best of our knowledge, studies examining the perspective of “whether the performer's subjectivity matches the physical variables” are rare. Therefore, we asked participants for a subjective assessment of the putting immediately after each putt, in a situation where they were not given visual information about the outcomes. That is, we clarify what kind of putts golfers perceive as poor (good) performances, how golfers estimate the outcomes, and what characteristics the putts actually (physically) have by comparing the estimated and actual average errors from the target. The second purpose is to quantitatively evaluate the performer's subjectivity generated by the ball's impact and to compare the kinematics and impact force of the putter-head based on those evaluations. We clarified the characteristics of the sense of distance among people with different skill levels. Note that the metrics focused on the first and second aims were different. We assume that amateurs have a gap between subjectivity and physics, and experts have no gap between them.

Based on the above, the present study recruits professional tour golfers and intermediate amateur golfers and investigates their putter-head kinematics and impact forces. The purpose of the present study is two-fold: first, to clarify whether kinematic variation of the putter head is correlated with the error in estimating the outcome and, second, to quantitatively evaluate the performer's sensations of good and poor performance generated by ball's impact for a comparison of the kinematics and impact force of the putter head based on their assessment. We analyze the variables that have been thought to be involved in the control of distance in previous studies (11, 15, 16).

## Methods

### Participants

This study includes 12 professional tour and 12 amateur golfers with average ages of 32.0 ± 4.3 years and 47.3 ± 11.2 years, respectively, and average experience levels of 19.9 ± 5.0 years and 15.5 ± 9.2 years, respectively. The amateurs are intermediate players with an average handicap of 15.0 ± 1.3. All the participants are right-handed golf players. Prior to participation, all participants provided informed written consent after receiving a complete explanation of the study. All experimental procedures have been approved by the ethics committee of Iwate Universirty and conform to the principles of the Declaration of Helsinki.

### Task and apparatus

The task includes two putting distances (2.4 and 4.8 m). The distance patterns were presented to each participant in a random order within a set, which included a total of 40 putts that consisted of two sets; no distance information was explicitly conveyed to the participants. The goal of each participant is to stop the ball in the center of a target that was the size of a real hole (10.8 cm in diameter). They were asked to show their own assessment of the Visual Analog Scale (VAS) after each putt. They were also asked to place a marker at the estimated Final Ball Position (FBP) after marking the putting assessment. In the trials, the participants could not confirm the FBPs, and their hearing was not obstructed. Therefore, they could hear the sound of the ball impact. However, the participants could not hear the sound of the ball rolling due to the use of an artificial turf designed for putting.

**Figure 1:**
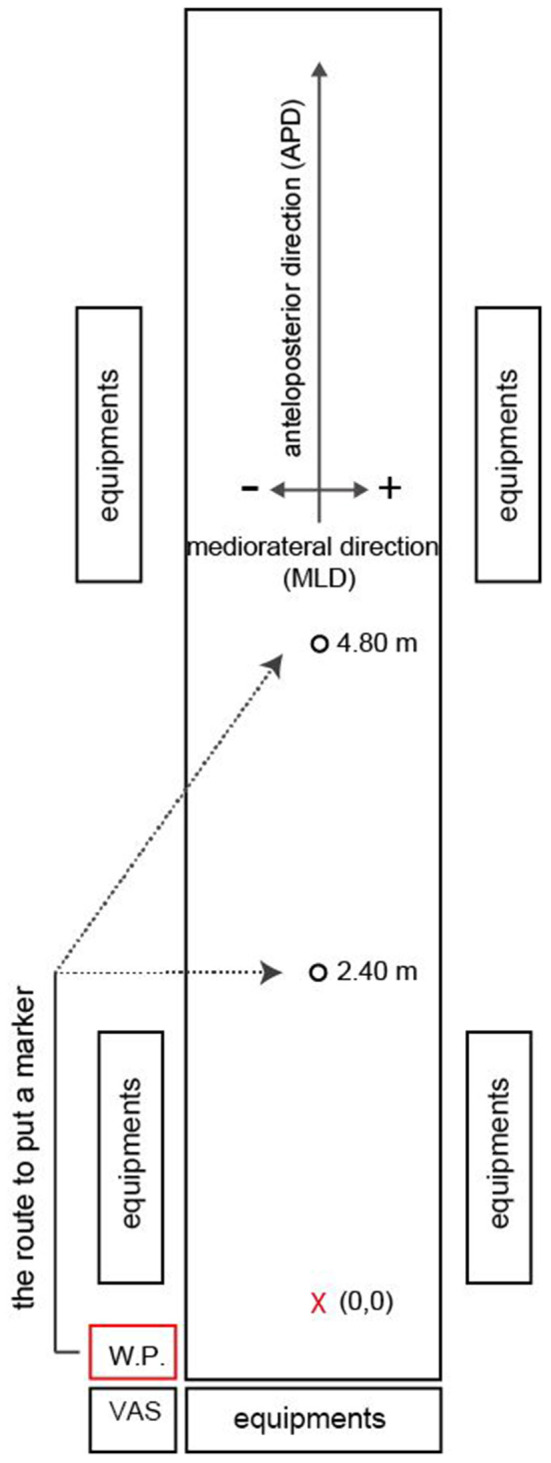
Experiment setting. The red X shows the initial position of the ball. W.P. indicates the waiting position of the participant during the trials. After hitting, participants turn to the desk and mark their rating on the visual analog scale (VAS). The reason participants walk the specified route is to avoid specifying the physical distance to the target. The targets are projected from the ceiling by two projectors.

**Figure 2:**
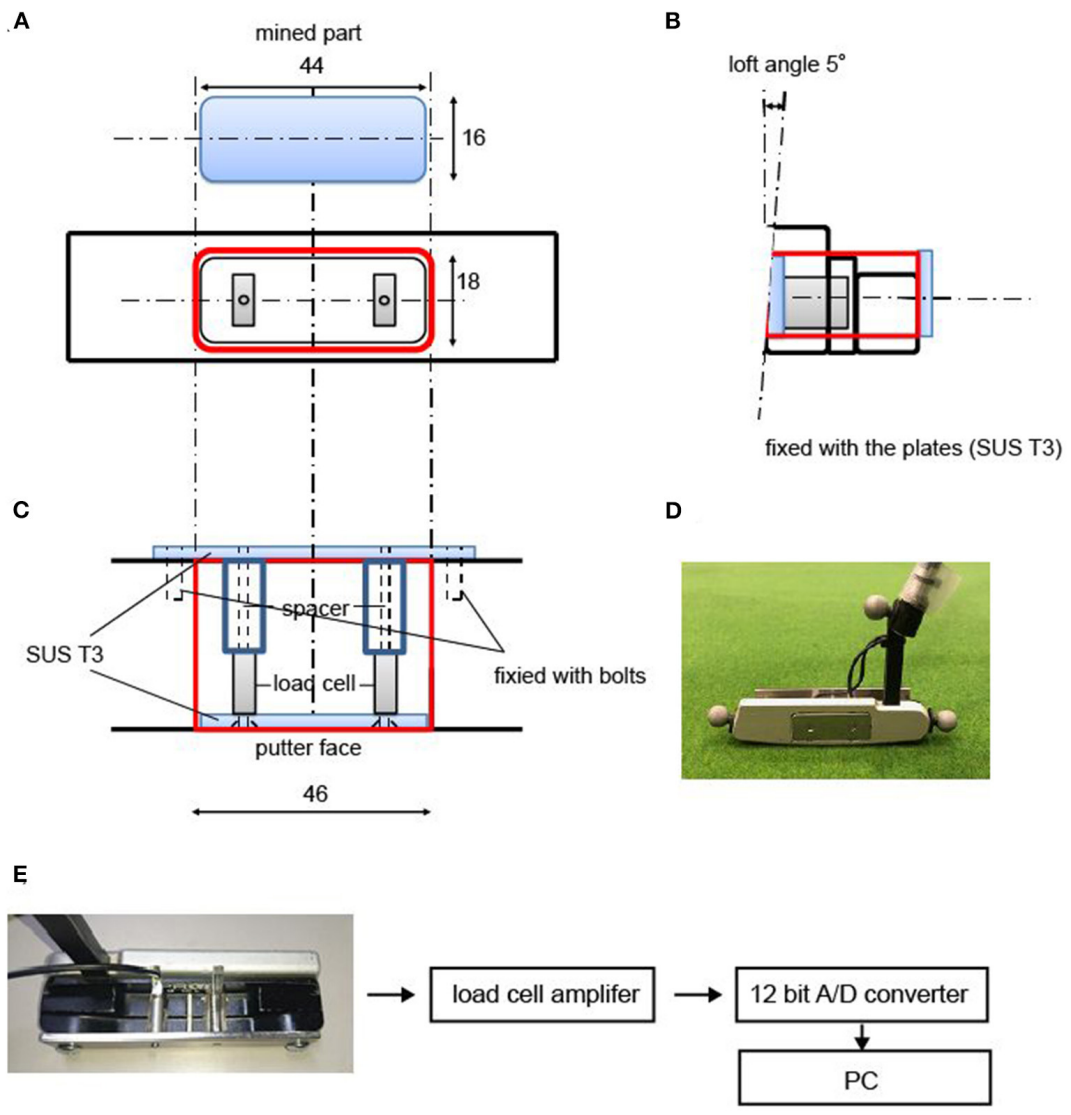
The putter equipped with the collision pressure measuring device. **(A)** shows the mined part of the putter face, the position, and the size of the equipped device. **(B)** shows an image from the side view of the device. **(C)** shows an image of the device from above. **(D)** is a photograph taken from the front of the putter equipped with the device used in the measurement. The markers for the motion capture system are affixed to the toe, heel, and neck of the putter head. **(E)** is a schematic diagram of the system. All numerical units in the figure are mm.

Figure 1 shows the experimental setup of this study. The target (light beam) is projected using two Offilio EB-1776W ceiling-mounted projectors for each target (Epson Corporation; Nagano, Japan) onto a single stretched (9.80 m long × 1.82 m wide) layer of artificial turf that was designed for putting practice (Superbent, Newtons Inc.; Kochi, Japan). The putting mat is laid on a wooden platform (9.80 m long × 1.82 m wide × 0.23 m high) and is set flat. Additionally, the participants wore a cap and glasses that limited their field of view to provide a 35-cm field ahead of the ball if they do not rotate their heads in the direction of the target (16). The VAS consists of a 10 cm one-dimensional straight line that could be entered by moving the cursor to the bottom edge for “very poor performance” and to the top edge for “the best performance”. A program using the Dasylab Ver.2016 data acquisition software (National Instruments; Austin, TX, USA) is used to calculate the VAS, which is scored at the distance (mm) from the bottom edge; the highest score is 100 and the lowest score is 0 (to the first decimal place). All participants use the same putter (SB-01HB, PRGR Corp., Yokohama, Japan) and balls (Srixon Z-Star XV, Dunlop Sports Co., Ltd., Hyogo, Japan). The putter has been converted into a collision-pressure measuring device [AO-50N, Applied Office Co., Ltd., Tokyo, Japan (Figures 2A–D)]. The signal from the strain-gauge type load cell embedded in the putter head is taken out as the voltage output by the load cell amplifier (rated capacity; 50N), and it is sampled at 10 kHz using a measurement computing 12-bit A/D converter (Figure 2E). Specifically, with the 50 N load cell, the load cell amplifier can be accurate up to 0.01 N. The A/D converter has a resolution of 0.0244 N. The recordings of the start and end operations of the putter collision pressure device have been synchronized with the optical motion capture system.

Putter head kinematics, FBPs, and estimated FBPs have been recorded using nine optical motion-capture cameras (OptiTrack Prime13; Acuity Inc., Tokyo, Japan) operating at 240 Hz. Additionally, 12-mm markers are attached to the toe, heel, and neck of the putter head to digitize the positions of the putter. The root mean square errors of both the static and dynamic calibrations are < 0.5 cm in the range of play (stroke areas), whereas they were < 1.5 cm in other locations (FBP) during all sessions.

### Procedure

After the participants provided their informed written consent, they received the following explanation from the experimenter. “From now on, I am going to ask you to putt 40 balls. After hitting 20 balls, take a break of 5 min and hit another 20 balls. When putting, there is no time limit or limit on the number of practice swings. Please wear glasses and caps to limit your field of view. You won't be able to see where the ball has stopped when you hit during the actual measurement. After hitting the ball, please turn on the opposite side without turning your head in the hitting direction. At this time, please keep in mind you're feeling about the putt and the image of where the ball has stopped. Then, go down the putting platform and enter your assessment of the putt using this tablet. After that, when I signal you, you will walk to the estimated final ball position and place a marker.” After this explanation, the experimenter adjusted the range of their field of vision to 35 cm ahead of the ball. This range was checked on occasions when the trial was paused (i.e., at breaks). The experimenter also explained to them how to enter their assessment on the VAS system and the route to place a marker (Figure 1). The participants were also briefed on how to handle the putter equipped with a collision-pressure measuring device. After hitting the ball, there was a waiting time of approximately 40 s before the participants had to place the marker (estimated FBP) in which the participants evaluated the trial. Information about these workflows was displayed in their waiting positions (see also Figure 1) so that they understand these workflows fully.

All the participants practiced 10 putts to the 2.4 m target and then practiced 10 putts to the 4.8 m target, or practiced the opposite pattern for a familiar session. At this time, they were able to confirm FBPs. Thereafter, the participants practiced 10 times (five balls in each target) to check the actual FBP after estimating it by facing the opposite side of the hitting direction after hitting the ball. To sum up, each participant hit a total of 71 balls in the familiar and test sessions. The order of target presentation at this time was random. Finally, one ball was used for each target in accordance with the experimental procedure.

The target was immediately erased after the ball impact. The ball hit by a participant was retrieved after capturing the FBP, and the target was lit again at the same position. The participant then placed a marker on the estimated FBP. In addition, the experimenter confirmed whether the participant turned his head in the hitting direction after ball impact in all trials. We confirm that none of the participants' heads turned to the target after ball impact in all the experimental trials.

### Dependent variables

#### Kinematics

All digitized data were smoothed with a fourth-order Butterworth filter (5-Hz cut-off) based on the root mean square of the residual error between the original and smoothed data (17, 18). The putting movement was divided into the backswing, downswing, ball impact, and follow-through phases (19, 20). The ball travel distance [i.e., the anteroposterior direction (APD), see also Figure 1] is highly dependent on the impact velocity (10, 15, 21). Since the main purpose of this study is to examine the sense of distance of the participants, we do not analyze the mediolateral direction (MLD), which is related to the orientation of the putter face during ball impact and the swing trajectory (22). According to previous research [e.g., Hasegawa et al. (9, 23)], the peak velocity is substituted as impact velocity because the impact velocity occurs immediately after the peak velocity. The measurement frequency was 240 Hz in this study, and the peak velocity was calculated instead of the impact velocity because the time resolution was insufficient to define the impact duration. The peak velocity targeted the component of the APD. The vertical and MLD components are not included it. We calculated the amplitude (i.e., downswing amplitude) and movement time (i.e., downswing time from the top of the swing to the impact) and the maximum acceleration based on a previous study (11). The midpoint between the toe and heel of the putter head is calculated to analyze the kinematics of the putter head. In addition, to confirm whether participants could hit the ball equipped with the collision-pressure measuring device, we calculated the estimated value of the ball's center position at the time of ball impact. We define how the rigid body of the putter (i.e., putter coordinates), as shown in Figure 2D, and the putter-ball impact position in the absolute coordinates was transformed into putter coordinates.

#### Impact force

**Figure 3:**
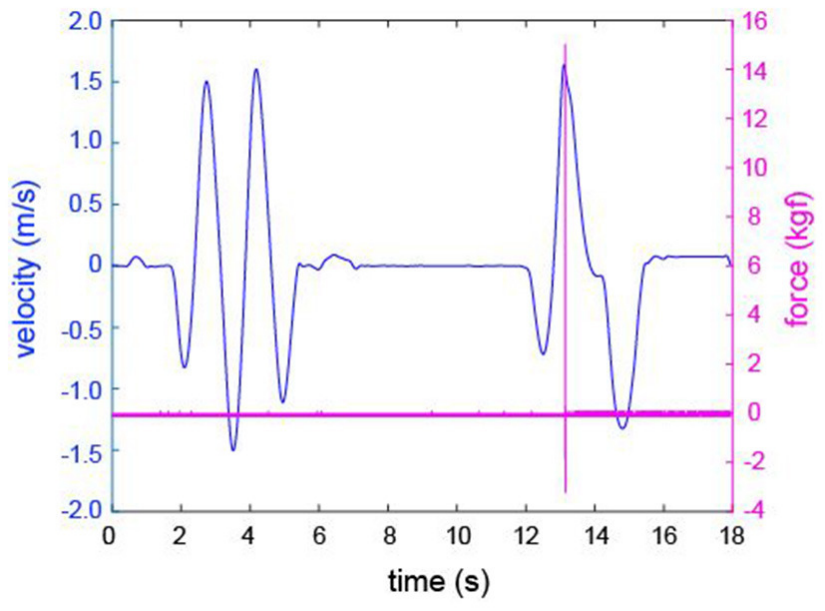
An example of data acquired by the motion capture system and the collision pressure measuring device. The left side y-axis and blue plots show the velocity of the putter head, and the right-side y-axis and pink plots show the value of the collision pressure measuring device. In the case of this figure, the actual hit was made after two practice swings. The peak value of force (around 13 s) indicates the impact. In this figure, the length of time is normalized. That is, the measurement frequencies (240 Hz and 10 kHz) of both devices are actually different.

Figure 3 shows an example of the data acquired by the motion capture system and collision-pressure measuring device. All the values obtained from the A/D converter were smoothed with a fourth-order Butterworth filter using a 3 kHz cut off frequency prior to calculation. We calculated the standard deviation (sd) of the values [kgf] of each trial for 1 second before the start of the swing. All participant's average values of the sd were −0.026 ± 0.037 kgf (2.4 m) and −0.024 ± 0.035 kgf (4.8 m). Then, the value from the moment it exceeds 10 sd to when it falls below the criteria was defined as the ball impact; the values between them (the number of contact times) were summed up, and 10 sd × the number of the contact time was subtracted from the total value. Finally, the value was multiplied by 9.8 to convert kgf to N and was then divided by the sampling frequency (10 kHz). We analyzed the impact force [N·S] known as impulse [N·S], which is a physical quantity.

#### Subjective putting assessment

The values marked by the participants were measured by the system as the distance from the bottom edge of the line (mm) and output to the first decimal place (see also 2.2. Task and apparatus).

#### Evaluation of actual final ball position

The actual FBP was divided into APD and MLD components, and the constant error (CE) of APD were determined. When the ball stopped at the target distance, the CE error was zero. The MLD component, which indicates the left-right direction errors, was not analyzed.

#### Evaluation of estimated final ball position

The estimated FBP was divided into APD and MLD components, and the APD of the estimated FBP was analyzed. To estimate an internal feedback error, as “the error of estimated FBP”, the actual FBP was subtracted from the estimated FBP in each trial and calculated as an absolute error. To evaluate the sense of distance of a participant, as “the CE of estimated FBP”, was calculated as the distance of the APD component from the center of the target to the estimated FBP of the participant, with positive and negative values. The MLD component was not analyzed.

### Statistics

To clarify the first purpose of the present study, we performed a simple regression analysis on each variable (the peak velocity, movement amplitude, movement time, maximum acceleration, and impact force) to extract the variable that best explained the ball's travel distance. In other words, we extracted the variable with the best model fit. The simple regression analysis was performed using all data (n = 960). In physics, the ball travel distance is determined by initial velocity, spin, and friction. The major determinant of the ball travel distance is initial ball velocity. And, the initial velocity is determined by applied force impulses. However, using measurement values, the resolutions and capabilities of the measurement system and the effects of noise must be considered. Pearson's correlation analysis was then performed between the coefficient of variation of the variable selected as the first variable in the simple regression analysis and the error of the estimated FBP for each group. As for the accuracy of outcome estimations, which has been discussed in previous studies (1, 2, 11), a two-factor mixed-design analysis of variance (ANOVA) was performed to explain the relationship between the two groups (professional, amateur) and the two distances (2.4, 4.8 m).

Next, to examine the second purpose of the study, for each individual, the subjective assessments were sorted in descending order and all dependent variables were sorted according to the VAS sequence. The top seven trials were defined as better, the bottom seven were defined as worse, and the average values for both were calculated as the representative value of each individual. A three-factor mixed-design analysis of variance (ANOVA) was then performed to explain the relationship between the two groups, the two distances, and the two subjectivities (better and worse) for each dependent variable. The results of the three-way ANOVAs are described as follows: second-order interactions (group × distance × subjective); first-order interactions (group × distance, group × subjective, and distance × subjective); and main effects. We calculated “f” values as effect-size indices for the ANOVAs (24). According to Cohen's (25) conventions, small (f = 0.10), medium (f = 0.25), and large (f = 0.40) effect sizes were reported. All data were analyzed using PASW Statistics (ver. 18.0; IBM Japan Ltd., Tokyo, Japan). The alpha level of significance was set to *p* < 0.05, but statistical results with effect size greater than medium (f = 0.25) were also mentioned.

## Results

### The results of simple regression analysis of each dependent variable for ball travel distance

Table 1 shows the results of simple regression analysis using each explanatory variable, with the ball travel distance as the response variable. All explanatory variables had significant relationships with the ball travel distance. According to the regression coefficients, we found that the explanatory variable with the best fit was the peak velocity. Therefore, we used the peak velocity for subsequent analyses.

**Table 1:** Results of simple regression analysis of each dependent variable for ball travel distance.

	**r^2^**	**Std. error**	***t* value**	***p* value**
Peak velocity	0.78	0.64	58.25	0.00
Impact force	0.75	0.67	54.28	1.59E-294
Movement amplitude	0.37	1.08	23.59	2.40E-97
Maximum acceleration	0.20	1.21	15.32	1.41E-47
Movement time	0.03	1.33	5.43	7.21E-08

R^2^ indicates regression coefficient. Std.error indicates standard error.

### The variability of movement and the error of the estimated outcome

**Figure 4:**
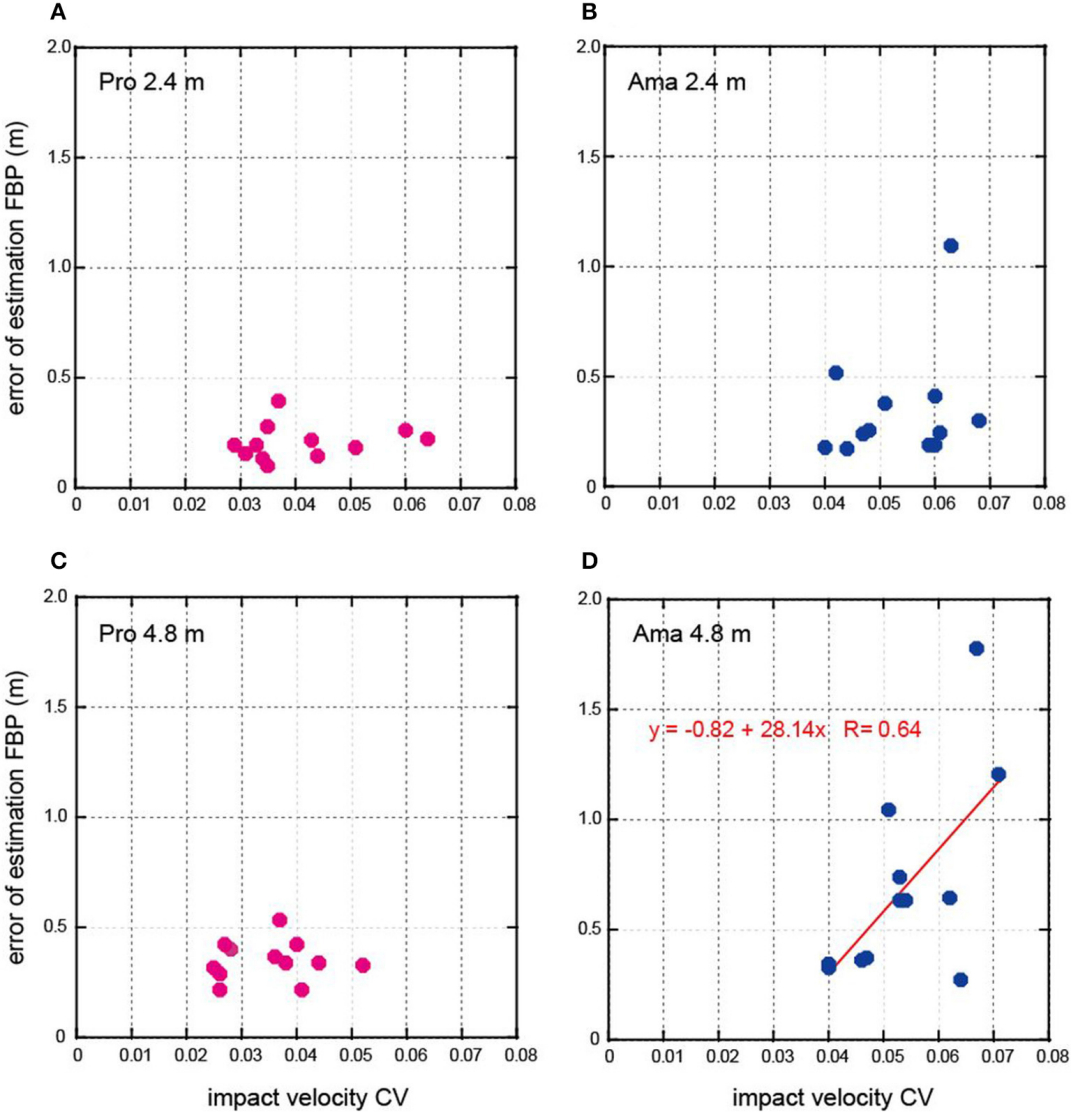
The results of the relationship between the peak velocity CV and the error of estimation FBP. **(A,C)** Show the results of professionals (Pro) for 2.4 m and 4.8 m putting, and **(B,D)** show the results of amateurs (Ama) for 2.4 m and 4.8 m putting.

Since it was confirmed that peak velocity was the strongest predictor of ball travel distance, we calculated the coefficient of variation (CV) of peak velocity. We then conducted Pearson's correlation analysis between peak velocity's CV and the error of the outcome estimations. This was carried out for each group and distance (Figure 4). There was a positive correlation between the two variables for amateur datasets of 4.8 m, r = 0.64, *p* < 0.05 (Figure 4D). However, no significant correlation was found between them for professional datasets (Figures 4A,B) and in the amateur 2.4 m putting (Figure 4C). Therefore, the peak velocity's CV is moderately related to the error of outcome estimation; although it depends on distance and skill level, our hypothesis is supported.

**Figure 5:**
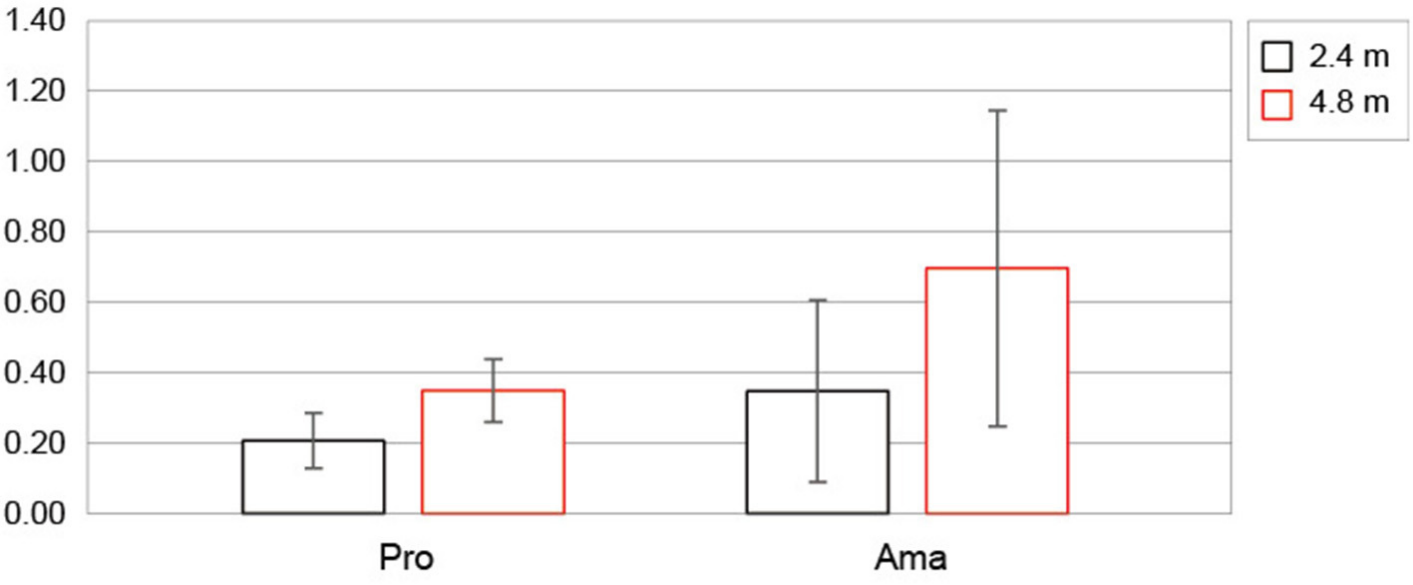
The error of estimated final ball position for each distance in each group. Error bars indicate ± 1 sd. Pro and Ama indicate professional and amateur, respectively. Unit is (m).

Figure 5 shows the average values of the error of the estimated FBP between the groups at each distance. The two-factor ANOVA results for the error in the estimated FBP revealed a significant interaction (F_1, 22_ = 6.22, p =0.021, f = 0.53, 1-β = 0.99). Simple effects testing indicated that the error of professionals for 2.4 m putting tended to be lower than that of amateurs (F_1, 22_ = 3.23, p =0.086, f = 0.38, 1-β = 0.54), and the error of professionals for 4.8 m putting was significantly lower than that of amateurs (F_1, 22_ = 6.93, *p* =0.015, f = 0.56, 1-β = 0.86). Also, the error of 4.8 m putting was higher than the error of 2.4 m putting in both professionals (F_1, 22_ = 5.79, *p* =0.025, f = 0.51, 1-β = 0.99) and amateurs (F_1, 22_ = 35.22, p = 5.68 × 10^−6^, f = 1.27, 1-β = 1.00).

### The characteristics of the sense of distance

#### Subjective putting assessment

Table 2 shows the statistics of the subjective putting assessments measured using VAS. The top seven trials were defined as better and the bottom seven were defined as worse; the average values for both were calculated as the representative value of each individual. The following analyses were performed based on these ratings.

**Table 2:** Statistics of subjective putting assessment.

	**Professional**				**Amateur**			
	**2.4 m**		**4.8 m**		**2.4 m**		**4.8 m**	
	**Ave**	**SD**	**Ave**	**SD**	**Ave**	**SD**	**Ave**	**SD**
Better	82.93	15.74	78.84	16.31	82.44	11.40	76.92	12.34
Worse	41.93	12.99	39.57	17.62	33.24	10.96	27.28	10.20
All	63.56	10.89	60.44	12.92	58.30	6.08	51.18	6.66

The highest value is 100 and the lowest value is 0. Sd represents the intra-individual standard deviations. The better shows the average of the top seven strokes in descending order. Worse shows the average of the bottom seven strokes of the VAS.

#### Estimated final ball position

**Figure 6:**
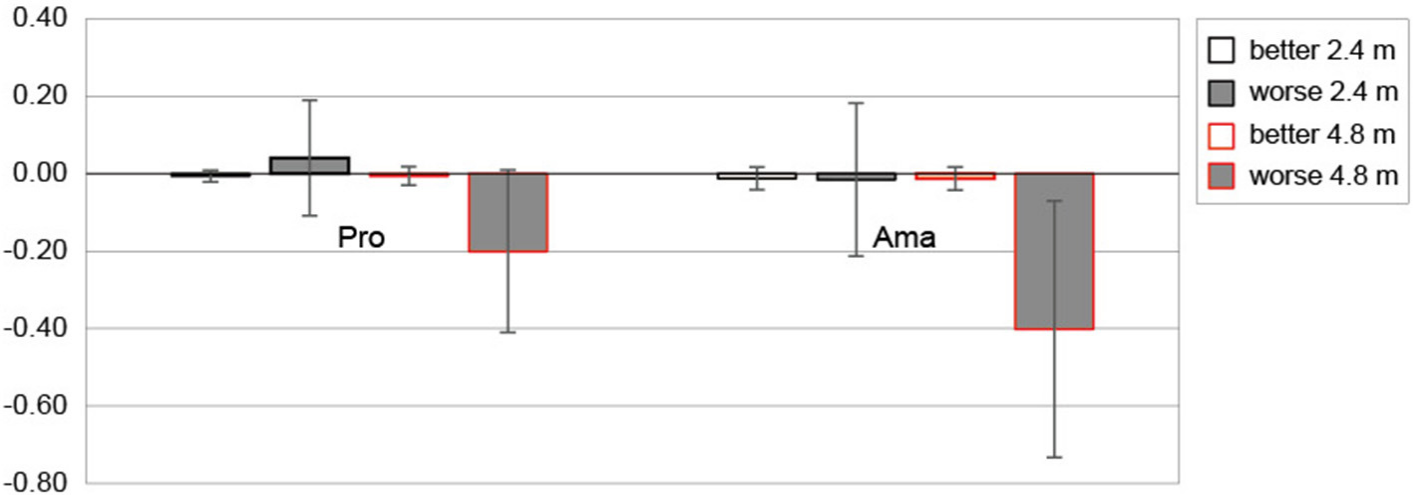
The constant error of estimated final ball position based on subjective putting assessment. Error bars indicate ± 1 sd. Pro and Ama indicate professional and amateur, respectively. Unit is (m).

Figure 6 shows the average values of estimated FBP (CE) between those rated as better and worse for both distances for each group. The three-factor ANOVA results for the estimated FBP revealed that the second-order interaction (group × distance × subjective) was not significant. However, a significant first-order interaction was observed (distance × subjective; F_1, 22_ = 32.64, p = 9.55 × 10^−6^, f = 1.22, 1-β = 1.00). Additionally, simple-effects testing indicated that the estimated FBP of 4.8 m putting rated as worse was shorter than the predicted FBP of 4.8 m putting rated as better (F_1, 22_ = 25.22, p = 5.00 × 10^−5^, f =1.07, 1-β = 1.00), and the estimated FBP of 4.8 m rated as worse was shorter than the estimated FBP 2.4 m rated as worse (F_1, 22_ = 34.39, p = 6.69 × 10^−6^, f = 1.25, 1-β = 1.00). Furthermore, the main effect of the group tended to be significant; the estimated FBP of amateurs tended to undershoot more than that of the professionals (F_1, 22_ =3.11, p =0.092, f = 0.38, 1-β = 0.54).

#### Actual final ball position

**Figure 7:**
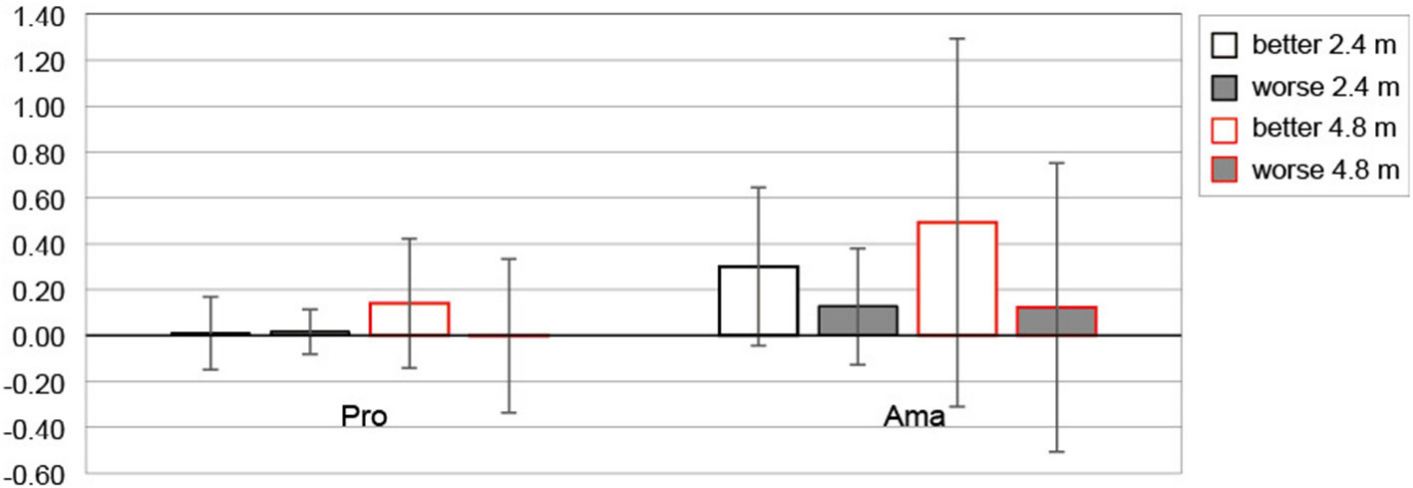
The constant error of the actual final ball position based on subjective putting assessment. Error bars indicate ± 1 sd. Pro and Ama indicate professional and amateur, respectively. Unit is (m).

Figure 7 shows the average values of CE between those rated as better and worse for both distances for each group. The three-factor ANOVA results for CE revealed that second-order interaction was not significant. However, a significant first-order interaction was observed (group × subjective; F_1, 22_ = 5.77, *p* = 0.025, f = 0.51, 1-β = 0.99). Additionally, simple-effects testing indicated that amateurs' CE rated as better was larger than the amateurs' CE rated as worse (F_1, 22_ = 20.92, *p* = 1.49 × 10^−4^, f = 0.98, 1-β = 1.00), and the amateurs' CE rated as better tended to be larger than the professionals' CE rated as better (F_1, 22_ = 3.52, *p* = 0.073, f = 0.40, 1-β = 0.96). Another interaction was observed (distance × subjective; F_1, 22_ = 6.88, *p* = 0.016, f = 0.56, 1-β = 0.99). Simple-effects testing indicated that the CE of 4.8 m putting rated as better was larger than the CE of 4.8 m putting rated as worse (F_1, 22_ = 17.30, *p* = 4.09 × 10^−4^, f =0.89, 1-β = 1.00), and the CE of 2.4 m putting rated as better tended to be larger than the CE of 2.4 m putting rated as worse (F_1, 22_ = 3.89, *p* = *0.061*, f = 0.42, 1-β = 0.98). Also, the CE of 4.8 m putting rated as better tended to be larger than the CE of 2.4 m putting rated as better (F_1, 22_ = 3.70, *p* = 0.067, f = 0.41, 1-β = 0.97).

#### Peak velocity

**Figure 8:**
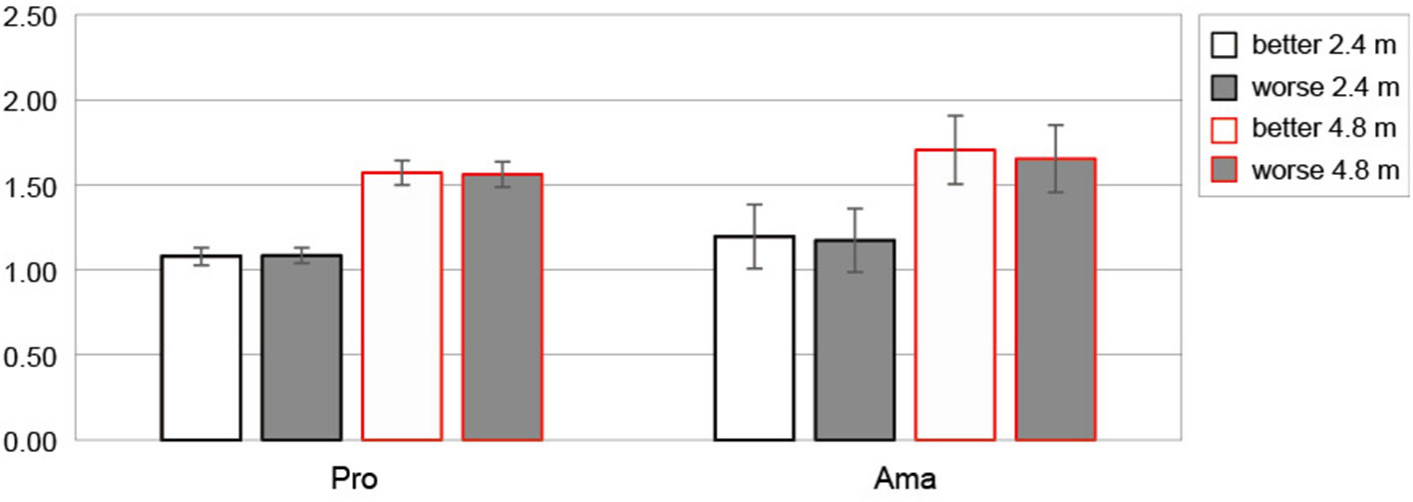
Average value of peak velocity based on subjective putting assessment. Error bars indicate ± 1 sd. Pro and Ama indicate professional and amateur, respectively. Unit is (m/s).

Of the many dependent variables, we analyzed peak velocity that explained the ball travel distance (see also Section The results of simple regression analysis of each dependent variable for ball travel distance). Figure 8 shows the average values of peak velocity between those rated as better and worse for both distances for each group. The three-factor ANOVA results for peak velocity revealed that the second-order interaction was not significant. However, a significant first-order interaction was observed (group × subjective; F_1, 22_ = 6.86, *p* = 0.016, f = 0.56, 1-β = 0.99). Simple-effects testing indicated that the peak velocity of amateurs rated as worse was lower than that of amateurs rated as better (F_1, 22_ = 15.37, *p* = 7.32 × 10^−4^, f = 0.84, 1-β = 1.00), and the peak velocity of amateurs rated as better was larger than that of professionals rated as better (F_1, 22_ = 4.66, *p* = 0.042, f = 0.46, 1-β = 0.70). In addition, other significant first-order interactions were observed (distance × subjective; F_1, 22_ = 4.65, *p* = 0.042, f = 0.46, 1-β = 0.99). Simple-effects testing indicated that the peak velocity of 4.8 m putting rated as better was larger than the peak velocity of 4.8 m putting rated as worse (F_1, 22_ = 11.11, *p* = 0.003, f = 0.71, 1-β = 0.99). Further, the peak velocity rated as worse and better were different depending on distances (2.4 m: F_1, 22_ = 1,741.91, *p* = 1.92 × 10^−22^, f = 8.90, 1-β = 1.00, 4.8 m: F_1, 22_ = 1,179.69, *p* = 1.31 × 10^−20^, f = 7.32, 1-β = 1.00); the peak velocity rated as worse was lower than the peak velocity rated as better.

To confirm whether participants could hit the ball equipped with the collision-pressure measuring device, we calculated the estimated value of the ball's center position at the time of ball impact. From the analyses, we confirmed that the center position of the ball at the time of the ball collision was inside the range of the collision-pressure measuring instrument (see Supplementary material 1.3.4, Supplementary Figure 1, Supplementary Table 1).

## Discussion

A previous study explained that a sense of distance may be learned and expressed through three skills: motor control of the putter head, perception of the impact force, and prediction of the putter head (11). The first purpose of the present study is to determine whether the variability in putter-head kinematics would have a strong correlation with the error of prediction outcome, which has remained an issue in previous research. The second purpose is to quantitatively evaluate the performer's sensations of good and poor performance generated by ball impact and to compare the kinematics and impact force of the putter-head based on those evaluations.

We analyzed the kinematic variables and impact forces and conducted simple regression analysis to determine the strongest predictor of ball travel distance (Table 1). According to the regression coefficients, the peak velocity was the strongest predictor for the ball travel distance. Based on the results, the CV of the peak velocity was calculated, and we found a moderately positive correlation between the two only in amateurs at 4.8 m putting (Figure 4). This suggests that motor control ability interacts with the error of outcome estimation, although it depends on skill level and distance to the target. In fact, this relationship was not confirmed in a previous study investigating the sense of distance using the golf-putting task (11).

We discuss the differences between the previous study and our results as follows: First, the errors in outcome estimation were small at shorter distances. Movements to a distant target are larger than those to a near target, so the variability of movement also increases (12, 26). Based on the results of a previous research (2) and our study, the errors of outcome estimations were also larger for longer distances than for shorter distances. The distances used in the previous study were 1.2 m, 2.4 m, and 3.6 m (11), and in our study as well, the relationship between motor control ability and the error of estimation outcome was not clear at 2.4 m putting. Second, the difference between previous research and our research is in the skill level of the participants. The participants of the previous study were advanced amateurs and novices. For novices, movement variability is large, and technically and cognitively immature, as reported in previous studies [e.g., (27–29)]. Therefore, the task of estimating outcomes may be difficult for novices. On the other hand, the higher the skill level, the smaller the variation in movement and the smaller the estimation error; therefore, it may be difficult to detect the relationship. Or, from another perspective, it might indicate that the variation of one movement variable does not directly affect the error of outcome estimation. Experts, such as tour professionals of golf, could compensate for the disturbance in one variable by the fine adjustment of the other variables (30, 31). In the future, it may be necessary to consider the variability and compensation of expert's motor control to clarify the relationship between motor control variability and estimation error.

In addition, regarding the error of estimation outcomes (Figure 5), several studies have demonstrated that the high-skilled group should estimate the outcomes of their performance more accurately than the low-skilled group (1, 2, 11), and the results are similar in the present study as well. Discreate and closed skills, such as golf putting, require the development of mental representations, and experts acquire more refined internal representations, which allows them to achieve more accurate and consistent outcomes (1, 2). According to motor learning theory, human motor skills are acquired by repeating three things: planning and preparation for the skill, skill execution and error detection between the expected results from the plan, and the actual skill performance (32). In addition, motor learning has been associated with systematic changes in proprioception (33–35) and generates accurate movements, improving sensory acuity (35). Based on these results, we tried the following attempts to increase our understanding of the sense of distance that humans have acquired through learning.

We investigated how putting that was subjectively rated as better by the participants and putting that was rated as worse differed depending on their skill level (Table 2). According to the results of the estimated FBP (Figure 6), it was clear that the output estimations for putting, which were evaluated as better, were close to 0 (close to the target distance) for both professionals and amateurs. Since the goal of the participants was to deliver the ball closer to the target, it is presumed that the trials in which the performer feels “good” are inseparable from the trials in which the ball is estimated to have almost reached the target. Further, especially at 4.8 m putting, there was a significant difference between better and worse putting ratings, and both groups estimated an undershoot. Furthermore, the statistical results also showed that amateurs tended to estimate more undershoots in FBP of 4.8 m putting than professionals. On the other hand, from the CE of actual FBP analysis, as shown in Figure 7, neither group showed an undershoot, especially in amateurs. We also found that the CE rated as better was larger than that rated as worse at both distances in both groups. In addition, the amateurs differed depending on the subjective evaluation, and the CE rated as better was larger than the CE rated as worse. In other words, amateurs rated the overshoot performance better. In addition, the CEs of amateurs that were evaluated as better tended to be larger than those of professionals. Summarizing the results of outcome estimations and the actual FBP, the estimated FBP for the putting that was rated as worse was the trial that participants felt was an undershoot. This tendency was especially noticeable in the amateur results for 4.8 m putting distance. However, such undershoot putting was not observed in the actual FBP, and the putting that amateurs rated as better was overshoot. Below, we discuss the results of the peak velocity, and give our suggestions obtained thus far.

We analyzed the peak velocity that explain ball travel distance. The peak velocity of the amateurs differed depending on the subjective evaluation, the peak velocity of the better rating was higher than that of the worse rating, the peak velocity of the better rating differed depending on the group, and the peak velocity of the amateurs' better rating was faster than that of the professionals' (Figure 8). Thus, kinematics also showed that our suggestion mentioned above, that is, the amateurs' sense of distance, was shifted above the target.

Why does the amateurs' sense of distance shift upward toward the target? Gray et al.'s (1) study reported that experts tend to predict better a performance, whereas novices tend to predict a worse performance, which might have been the reason for the shift. We surmise that the amateurs were afraid of undershoots caused by failure to hit the ball properly. It has been pointed out that non-experts cannot catch the ball in the center of the putter head compared to experts (22). This result was also confirmed by the additional analysis in the present study, in which amateurs were hitting the ball more with the toes of the putter head (see Supplementary material 1.3.4, Supplementary Figure 1, Supplementary Table 1). Hitting a ball at the center of the putter head creates a stable ball movement distance (8). Therefore, amateurs may require higher velocity than professionals. It has been confirmed that amateurs have a higher peak velocity than professionals, even at the same target distance (21). We believe that the amateurs were hitting at a higher velocity to compensate for the risk of undershoot.

However, we could not find the characteristics of a professional's sense of distance in the present study. We believe that this shows that there are no extreme differences in the mistakes made by the experts. The professionals also had less accuracy in their outcome estimations when they putt for a longer distance, which means that there was a discrepancy between what was expected and what really was, but not as noticeably as for amateurs. However, there was a clear difference between the better and worse subjective assessments quantified by the visual analog scale (Table 2). Therefore, it is possible that the measurement items and resolutions used in the present study could not distinguish the professionals' good feeling putts from the bad ones. It is also why peak velocity was chosen as the variable that best describes the ball's travel distance for the first purpose. The impact force should be chosen as the variable that best explains the ball's travel distance in physics. We calculated the impulse from the force (kgf). The discrepancy between measurements and theory of physics means that system performance or noise effects are present. That is, the impulse might be uncertain due to insufficient time and force resolution, and the effects of measurement noise. Otherwise, the difference in movement at the professional level is extremely small and can be buried in measurement noise. To gain a better understanding of the excellent sense of distance observed in human performance, it is necessary to solve these problems in the future.

## Conclusion

We recruited professional and amateur golfers and conducted an experiment to investigate their sense of distance using the golf-putting task. As confirmed in previous studies, professionals estimated the final ball position more accurately than amateurs did, and their mental representation was found to be superior. We found a moderate correlation between the variation in peak velocity, which is an index of motor control ability, and the error of outcome estimation, especially at longer distances for amateurs. Furthermore, we tried to quantify the subjectivity of ball impact to understand the characteristics of a golfers' sense of distance. We then investigated the relationship between the physical results (movements and results) and subjectivity (better or worse). As a result, it became clear that the putts when amateurs felt better were actually overshooting, and that the amateurs' sense of distance shifted upward to the target. In other words, the subjectively “good putt” of amateurs was not physically good. However, there were no clear characteristics for professionals, and there was no clear difference between subjectivity and physics.

## Data availability statement

The datasets presented in this study can be found in online repositories. The names of the repository/repositories and accession number(s) can be found in the article/Supplementary material.

## Ethics statement

All experimental procedures were approved by the Ethics Committee of Iwate University, Morioka, Japan. The patients/participants provided their written informed consent to participate in this study.

## Author contributions

YH, AO, and KF conceived and designed the study. YH and AO conducted experiments. YH and KF analyzed the data. All authors contributed to the study and approved the final manuscript.

## Funding

This study was funded by the JSPS KAKENHI (Grant Nos. JP18K17818 and JP20H04075) and JST PRESTO (Grant No. JPMJPR20CA). The funders had no role in the study design, data collection and analysis, decision to publish, or manuscript preparation.

## Conflict of interest

The authors declare that the research was conducted in the absence of any commercial or financial relationships that could be construed as a potential conflict of interest.

## Publisher's note

All claims expressed in this article are solely those of the authors and do not necessarily represent those of their affiliated organizations, or those of the publisher, the editors and the reviewers. Any product that may be evaluated in this article, or claim that may be made by its manufacturer, is not guaranteed or endorsed by the publisher.
