# Supplementary material for: A sense of distance and movement characteristics of golfers tested without visual feedback of outcomes: Is a putt that feels subjectively good also physically good?
Source: Front Sports Act Living. 2022 Oct 25;4:987493. doi: 10.3389/fspor.2022.987493 (PMC9640950; doi:10.3389/fspor.2022.987493)
Supplement: Supplementary file 2 [file Data_Sheet_2.PDF]

Supplementary Material

Supplementary Text

1 Results

1.1 The results of simple regression analysis of each dependent variable for ball travel distance

Table 1 shows the results of simple regression analysis using each explanatory variable, with the ball travel distance as the response variable. All explanatory variables had significant relationships with the ball travel distance. According to the regression coefficients, we found that the explanatory variable with the best fit was peak velocity. Therefore, we used the peak velocity for subsequent analyses.

1.2 The variability of movement and the error of the estimated outcome

Since it was confirmed that peak velocity was the strongest predictor of ball travel distance in both groups, we calculated the coefficient of variation (CV) of peak velocity. We then conducted Pearson's correlation analysis between peak velocity's CV and the error of the outcome estimations. This was carried out for each group and distance (Figure 4). There was a positive correlation between the two variables for amateur datasets of 4.8 m, $r = 0.64$, $p < .05$ (Figure 4D). However, no significant correlation was found between them for professional datasets (Figure 4A, 4B) and in the amateur 2.4 m putting (Figure 4C). Therefore, the peak velocity's CV is moderately related to the error of outcome estimation; although it depends on distance and skill level, our hypothesis is supported.

Figure 5 shows the average values of the error of the estimated FBP between the groups at each distance. The two-factor ANOVA results for the error in the estimated FBP revealed a significant interaction ($F_{1,22} = 6.22$, $p = .021$, $f = 0.53$, $1\text{-}\beta = 0.99$). Simple effects testing indicated that the error of professionals for 2.4 m putting tended to be lower than that of amateurs ($F_{1,22} = 3.23$, $p = .086$, $f = 0.38$, $1\text{-}\beta = 0.54$), and the error of professionals for 4.8 m putting was significantly lower than that of amateurs ($F_{1,22} = 6.93$, $p = .015$, $f = 0.56$, $1\text{-}\beta = 0.86$). Also, the error of 4.8 m putting was higher than the error of 2.4 m putting in both professionals ($F_{1,22} = 5.79$, $p = .025$, $f = 0.51$, $1\text{-}\beta = 0.99$) and amateurs ($F_{1,22} = 35.22$, $p = 5.68\times10^{-6}$, $f = 1.27$, $1\text{-}\beta = 1.00$).

1.3 The characteristics of the sense of distance

1.3.1 Subjective putting assessment

Table 1 shows the statistics of the subjective putting assessments measured using VAS. The top seven trials were defined as better and the bottom seven were defined as worse; the average values for both were calculated as the representative value of each individual. The following analyses were performed based on these ratings.

1.3.2 Estimated final ball position

Figure 6 shows the average values of estimated FBP between those rated as better and worse for both distances for each group. The three-factor ANOVA results for the estimated FBP revealed that the second-order interaction (group × distance × subjective) was not significant. However, a significant first-order interaction was observed (distance × subjective; $F_{1,22} = 32.64$, $p = 9.55\times10^{-6}$, $f = 1.22$, $1\text{-}\beta = 1.00$). Additionally, simple-effects testing indicated that the estimated FBP of 4.8 m putting rated as worse was shorter than the predicted FBP of 4.8 m putting rated as better ($F_{1,22} = 25.22$, $p = 5.00\times10^{-5}$, $f = 1.07$, $1\text{-}\beta = 1.00$), and the estimated FBP of 4.8 m rated as worse was shorter than the estimated FBP 2.4 m rated as worse ($F_{1,22} = 34.39$, $p = 6.69\times10^{-6}$, $f = 1.25$, $1\text{-}\beta = 1.00$). Furthermore, the main effect of the group tended to be significant; the estimated FBP of amateurs tended to undershoot more than that of the professionals ($F_{1,22} = 3.11$, $p = .092$, $f = 0.38$, $1\text{-}\beta = 0.54$).

1.3.3 Actual final ball position

Figure 7 shows the average values of CE between those rated as better and worse for both distances for each group. The three-factor ANOVA results for CE revealed that second-order interaction was not significant. However, a significant first-order interaction was observed (group × subjective; $F_{1,22} = 5.77$, $p = .025$, $f = 0.51$, $1\text{-}\beta = 0.99$). Additionally, simple-effects testing indicated that amateurs' CE rated as better was larger than the amateurs' CE rated as worse ($F_{1,22} = 20.92$, $p = 1.49\times10^{-4}$, $f = 0.98$, $1\text{-}\beta = 1.00$), and the amateurs' CE rated as better tended to be larger than the professionals' CE rated as better ($F_{1,22} = 3.52$, $p = .073$, $f = 0.40$, $1\text{-}\beta = 0.96$). Another interaction was observed (distance × subjective; $F_{1,22} = 6.88$, $p = .016$, $f = 0.56$, $1\text{-}\beta = 0.99$). Simple-effects testing indicated that the CE of 4.8 m putting rated as better was larger than the CE of 4.8 m putting rated as worse ($F_{1,22} = 17.30$, $p = 4.09\times10^{-4}$, $f = 0.89$, $1\text{-}\beta = 1.00$), and the CE of 2.4 m putting rated as better tended to be larger than the CE of 2.4 m putting rated as worse ($F_{1,22} = 3.89$, $p = .061$, $f = 0.42$, $1\text{-}\beta = 0.98$). Also, the CE of 4.8 m putting rated as better tended to be larger than the CE of 2.4 m putting rated as better ($F_{1,22} = 3.70$, $p = .067$, $f = 0.41$, $1\text{-}\beta = 0.97$).

1.3.4 Peak velocity

Of the many dependent variables, we analyzed peak velocity that explained the ball travel distance (see also 3.1 in the main text). Figure 8 shows the average values of peak velocity between those rated as better and worse for both distances for each group. The three-factor ANOVA results for peak velocity revealed that the second-order interaction was not significant. However, a significant first-order interaction was observed (group × subjective; $F_{1,22} = 6.86$, $p = .016$, $f = 0.56$, $1\text{-}\beta = 0.99$). Simple-effects testing indicated that the peak velocity of amateurs rated as worse was lower than that of amateurs rated as better ($F_{1,22} = 15.37$, $p = 7.32\times10^{-4}$, $f = 0.84$, $1\text{-}\beta = 1.00$), and the peak velocity of amateurs rated as better was larger than that of professionals rated as better ($F_{1,22} = 4.66$, $p = .042$, $f = 0.46$, $1\text{-}\beta = 0.70$). In addition, other significant first-order interactions were observed (distance × subjective; $F_{1,22} = 4.65$, $p = .042$, $f = 0.46$, $1\text{-}\beta = 0.99$). Simple-effects testing indicated that the peak velocity of 4.8 m putting rated as better was larger than the peak velocity of 4.8 m putting rated as worse regardless of groups ($F_{1,22} = 11.11$, $p = .003$, $f = 0.71$, $1\text{-}\beta = 0.99$). Further, the peak velocity rated as worse and better were different depending on distances regardless of groups (2.4 m: $F_{1,22} = 1741.91$, $p = 1.92\times10^{-22}$, $f = 8.90$, $1\text{-}\beta = 1.00$, 4.8 m: $F_{1,22} = 1179.69$, $p = 1.31\times10^{-20}$, $f = 7.32$, $1\text{-}\beta = 1.00$).

To confirm whether participants could hit the ball equipped with the collision-pressure measuring device, we calculated the estimated value of the ball's center position at the time of ball impact. Supplementary Figure 1 shows the calculated ball center position at impact for all trials. Supplementary Table 1 shows the averages and standard deviations of these values. From these analyses, it is confirmed that the center position of the ball at the time of the ball collision was inside the range of the collision-pressure measuring instrument. For additional analyses, we performed a two-factor ANOVA (group × distance) on the ball center position (toe-heel and top-bottom components) to determine whether the impact position was different for each group (see supplementally text 1.3.4). The two-factor ANOVA results for the impact point of the toe-heel component (i.e., horizontal) revealed that the interaction was not significant. However, the main effect of group was significant ($F_{1,22} = 16.47$, $p = 5.23\times10^{-4}$, $f = 0.87$, $1-\beta = 0.99$). The impact point of amateurs was farther from the center of the putter head (on the toe side) than that of the professionals. On the other hand, the two-factor ANOVA results for the impact point of the top-bottom component (i.e., vertical) revealed that the interaction was significant ($F_{1,22} = 6.12$, $p = .022$, $f = 0.53$, $1-\beta = 0.99$). Simple-effects testing indicated that the impact point of professionals' 2.4 m putting was higher than that of professionals' 4.8 m putting ($F_{1,22} = 13.13$, $p = .002$, $f = 0.77$, $1-\beta = 0.99$, and the impact point of amateurs' 4.8 m putting tend to be farther from the center of the putter head (in the top side) than that of professionals ($F_{1,22} = 3.09$, $p = .092$, $f = 0.37$, $1-\beta = 0.52$).

2 Supplementary Figure

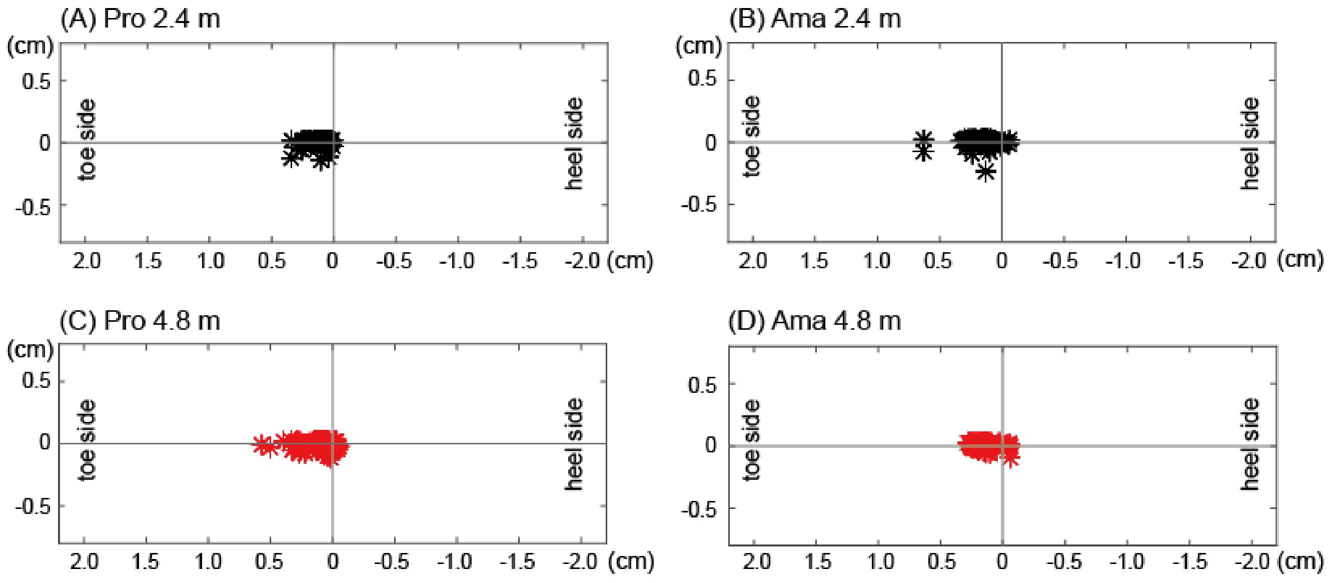

Supplementary Figure 1. All trials of impact point for each distance in each group. The asterisks indicate the estimated ball center position at ball impact. (A) and (C) show the results of professionals (Pro) for 2.4 m and 4.8 m putting, and (B) and (D) show the results of amateurs (Ama) for 2.4 m and 4.8 m putting. If the center of the ball is located at the center of the face, the asterisk is plotted (0,0). 240 putts are plotted at each distance for each group. The unit in this figure is centimeters. The outer frame in the figures show the size of the device (see also Figure 2 in the main text).

3 Supplementary Table

Supplementary Table 1. Averages and standard deviations of impact point.

	Professional				Amateur			
	2.4 m		4.8 m		2.4 m		4.8 m	
	average	sd	average	sd	average	sd	average	sd
toe-heel	0.096	0.031	0.098	0.033	0.152	0.039	0.145	0.026
top-bottom	0.014	0.009	0.007	0.014	0.015	0.007	0.015	0.007

Note. This table shows the mean and standard deviation in Supplementary Figure 1. Sd: standard deviation.
